# WhatsGNU: a tool for identifying proteomic novelty

**DOI:** 10.1186/s13059-020-01965-w

**Published:** 2020-03-05

**Authors:** Ahmed M. Moustafa, Paul J. Planet

**Affiliations:** 1 Division of Pediatric Infectious Diseases, Children’s Hospital of Philadelphia, Philadelphia, PA 19104 USA; 2 Department of Pediatrics, Perelman College of Medicine, University of Pennsylvania, Philadelphia, PA 19104 USA; 3 Sackler Institute for Comparative Genomics, American Museum of Natural History, New York, NY 10024 USA

**Keywords:** *S. enterica*, *S. aureus*, *P. aeruginosa*, *M. tuberculosis*, Panallelome, Pangenome, Compression, Microbial genomics, blastp

## Abstract

To understand diversity in enormous collections of genome sequences, we need computationally scalable tools that can quickly contextualize individual genomes based on their similarities and identify features of each genome that make them unique. We present WhatsGNU, a tool based on exact match proteomic compression that, in seconds, classifies any new genome and provides a detailed report of protein alleles that may have novel functional differences. We use this technique to characterize the total allelic diversity (panallelome) of *Salmonella enterica*, *Mycobacterium tuberculosis*, *Pseudomonas aeruginosa,* and *Staphylococcus aureus*. It could be extended to others. WhatsGNU is available from https://github.com/ahmedmagds/WhatsGNU.

**Electronic supplementary material:**

**Supplementary information** accompanies this paper at 10.1186/s13059-020-01965-w.

## Introduction

With vastly reduced sequencing costs and the exponential growth of public genomic databases, scalable tools are needed to categorize and classify new sequences and measure genomic novelty [1]. Currently, 30 of the microbial species in NCBI have more than 1000 assemblies each, and the top 7 have more than 10,000 assemblies each [2].

Traditional methods for identifying new polymorphisms in a genome rely on a single reference sequence for comparison, an approach that is limited because it can only describe differences from the reference and cannot tell whether the identified polymorphisms are rare or widespread in the natural variation of the species. The use of multiple references might still ignore known variation, and using the entire database of available reference genomes becomes computationally intractable as databases grow.

One way to compress the information in large databases is to eliminate copies of redundant sequences that are exactly the same while retaining information about the genomes in which they are found, reducing databases to a fraction of their original size. Importantly, this method can also yield a simple count of the number of exact protein sequence matches (100% identity and coverage) for any given protein allele in the database. This simple count, which we call the gene novelty unit (GNU) score, provides a useful metric that can be used in multiple ways in comparative analysis, some of which we demonstrate here. The GNU score for each protein is inversely proportional to novelty. Proteins with a low GNU score are infrequent in genomes in the database. A GNU score of zero means that there is no match, the first known allele of its kind. A high GNU score mean that this allele is well represented in the database and is likely to be a highly conserved protein. In essence, the GNU score is of interest because it describes what we “know” already about a protein variant across the entire database; it can be used to gauge the novelty of a newly observed allele and the overall amount of novelty in a proteome.

Several tools have been developed to assess genetic variant frequencies in human genomic databases using nucleotide sequences [3–8], but we are not aware of any tools for either microbial or eukaryotic genomes that assess protein allele frequency in public or private databases by constructing a panallelome of an entire species.

We developed the WhatsGNU tool to quickly calculate the GNU score for each protein across a genome and provide a range of comparative graphs for publication quality figures. With the addition of information about the orthology of each allele, WhatsGNU can also be used to link pangenomic and panallelomic analyses.

## Results and discussion

The WhatsGNU toolbox is composed of four Python3 [9] scripts that (1) download GenBank genomes, (2) customize annotations with strain name and metadata, (3) compress all protein sequences to unique alleles, and (4) plot graphs of the results (Additional file 1). It accepts for analysis protein FASTA files of genomes that are produced by annotation tools such as Prokka [10] and RAST [11]. The database file could be derived from public databases such as GenBank [12, 13] or new, unpublished data.

**Fig. 1:**
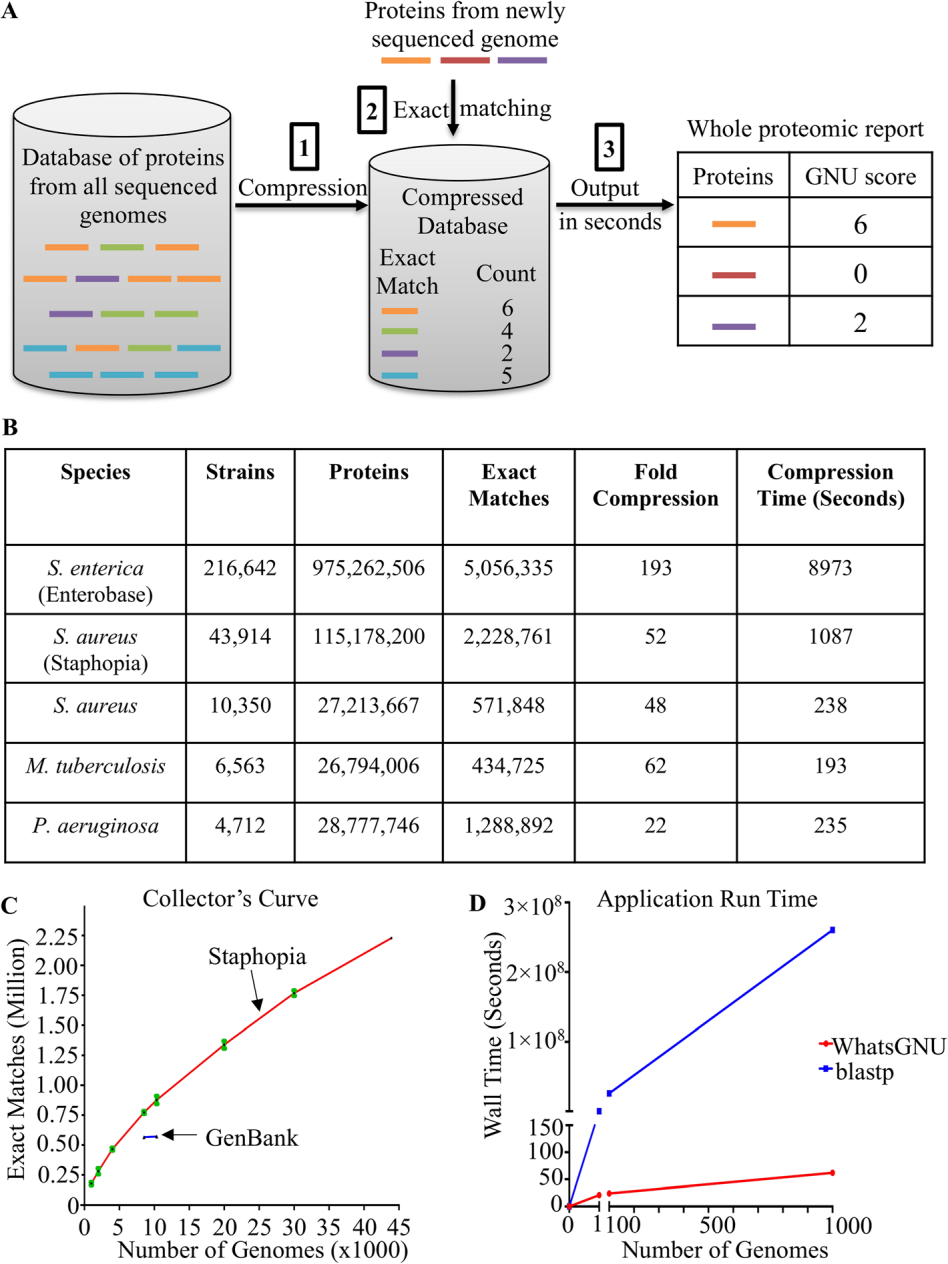
Workflow and performance of WhatsGNU. **a** Workflow for the WhatsGNU tool and its compression technique. The tool starts by compressing the database of proteins. The second step is to match each protein from a query genome to an exact match in the compressed database. The final step is to produce a report with a GNU (Gene Novelty Unit) score for each protein. **b** Compressed Databases available in WhatsGNU. **c** A collector’s curve expresses the number of exact matches (unique alleles) as a function of the number of genomes sequenced. The size of the panallelome of available genomes of *S. aureus* on GenBank and Staphopia were compared. The 1000, 2000, 4000, 8524, 10,350, 20,000, and 30,000 genomes from the 43,914 *S. aureus* genomes available on Staphopia were randomly selected. The random sampling step was done three times, independently. The error bars are shown in green. **d** Effect of the number of isolates on the running wall time of WhatsGNU and blastp. Both WhatsGNU and blastp were used on a single CPU and 16 GB of RAM. The *S. aureus* database used for WhatsGNU was previously processed and serialized using the Python3 pickle module. The time needed to find exact matches for each of the 2893 proteins of *S. aureus* NCTC 8325 was noted for WhatsGNU and blastp. 1, 100, and 1000 copies of NCTC 8325 genome were used to evaluate the running time for WhatsGNU. For blastp, to reduce computational costs, the running time of one NCTC 8325 genome was multiplied by 100 and 1000, respectively. Running time would differ on desktops with different specifications. Blastp running time can be reduced by using multiple threads if more than one CPU is available

WhatsGNU can compress large bacterial genomic databases of 4000–10,000 genomes to nonredundant panallelomes in less than 4 min on a standard laptop processor. Two of the biggest available curated bacterial collections, 43,913 genomes in Staphopia [14] and 216,642 in Enterobase [15], took less than 20 min and 2.5 h, respectively (Fig. 1a, b and supplementary Table 1). Databases had approximately 20- to 190-fold compression with no information loss.

To better understand the performance of WhatsGNU compression with large numbers of genomes in the database, we generated a collector’s curve of the Staphopia database by randomly resampling the database and noting the size of the compressed panallelome. The resulting curve never plateaus suggesting that there is unsampled allelic diversity in *S. aureus*. Interestingly, when compared to databases available at GenBank, Staphopia shows higher numbers of unique alleles at similar numbers of sampled genomes suggesting that the GenBank database may have more sampling bias (Fig. 1c and supplementary Table 2).

Once a compressed database is loaded, running a WhatsGNU report on any new single genome takes less than 1 s (Additional file 4). The detailed proteomic output of WhatsGNU gives a GNU score for each protein in the genome and any other appended metadata (Additional file 4). Genomes can be batched for analysis, and experiments on a single processor computer showed that 100 and 1000 *S. aureus* genomes could be analyzed by WhatsGNU in 24 and 62 s, respectively, while blastp [16] took 3 days to analyze one genome (Fig. 1d). The numbers of exact matches reported by WhatsGNU were identical to blastp (Additional files 1 and 4).

One potential limitation of the GNU score is that completely “novel” variants may in fact be due to sequencing errors or incomplete assemblies that produce truncated genes. In a test set of 16 *S. aureus* genomes of varying quality, we noted a strong linear correlation between the number of proteins with GNU score of zero and the number of contigs for each assembly (*P* < 0.0001, R^2^ = 0.9544, Fig. S1). This strong relationship suggests that proteins with GNU = 0 should be treated cautiously, with confirmatory sequencing if necessary, and that WhatsGNU will perform best with high-quality sequences. Low, non-zero, GNU scores (e.g., 1–10) may be less likely to be sequencing errors since they have been sequenced in other genomes.

**Fig. 2:**
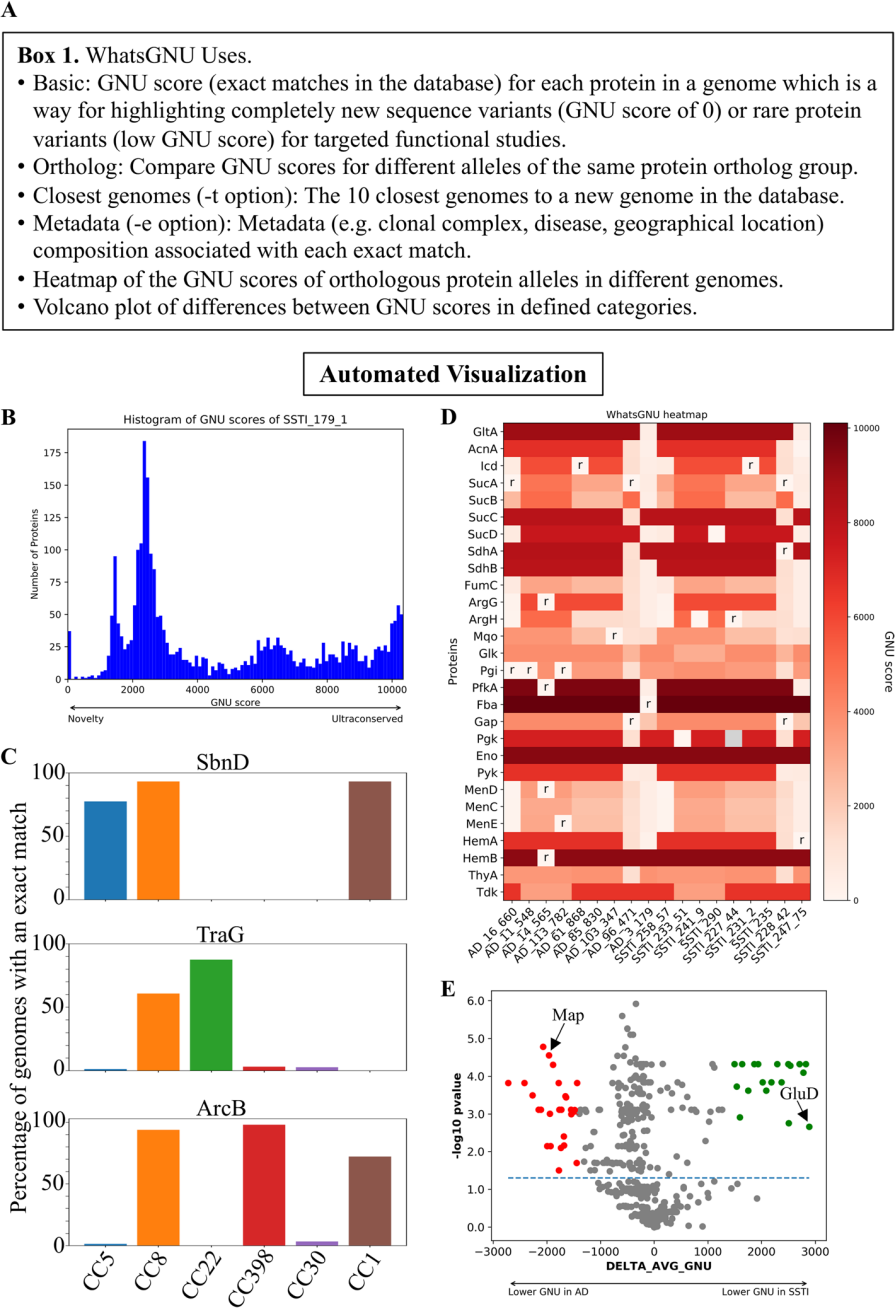
Visualization methods of WhatsGNU. **a** Box showing some potential WhatsGNU uses and options. **b** A histogram of GNU scores of a clinical-CC8-USA300 *S. aureus* genome “SSTI_179_1”. **c** Percentage of genomes of clonal complexes (CC) 5, 8, 22, 398,30, and 1 with an exact match for three proteins, SbnD (staphyloferrin B export MFS transporter), TraG (Transfer complex protein), and ArcB (ornithine carbamoyltransferase) from the same genome used in **b**. **d** A heatmap of GNU score for key components of the TCA cycle, the glycolytic pathway, and terminal components of the electron transport chain in eighteen different clinical *S. aureus* isolates. Proteins are listed on the left and isolates numbers on the bottom. In the case of annotated cells, ‘r’ refers to ortholog variant rarity index (OVRI) scores that are less than 0.045. This can be interpreted as an indication that GNU scores this low or lower are very rare in this ortholog group. **e** Volcano plot showing proteins with a lower average GNU score in a case group (atopic dermatitis) compared to a control group (soft and skin tissue infection). Proteins with lower average GNU score in the AD case group of 18 CC8 *S. aureus* isolates are shown in red. Proteins with lower average GNU score in the SSTI control group of 49 CC8 *S. aureus* isolates are shown in green. The *P* value is from a Mann–Whitney–Wilcoxon test. A second volcano plot with *Y*-axis as OVRI is shown in supplementary figure 3. Example WhatsGNU reports are in the supplementary data

In addition to exact match compression and GNU score reports, WhatsGNU offers several other features (Fig. 2a). One such feature is the ability to quickly find the closest match genomes from the database by reporting those that have the highest numbers of exact matches to the query genome. This could be useful for selecting closely related genomes for reference-based comparisons and offers a preliminary classification similar to a complete genome multilocus sequence type. In a comparison with the existing tool Mash [17], this functionality produced an identical list of the top 10 genome hits, but WhatsGNU was 2.5 times faster (Details in Additional file 1).

The GNU score can be a useful metric for comparative genomic analysis that gives insight into the distribution of allelic diversity across a genome. In the example histogram (Fig. 2b, Fig. S2 and Additional file 4) of proteins in a single genome, the first peak of 37 proteins (GNU< 100) represents the potential novelty in the genome of rarely seen variants, while the peaks around GNU > 10,000 represent highly conserved proteins in the species. The three peaks around GNU scores of 1200, 1900, and 5000 represent alleles shared with specific clades or lineages (USA300, CC8, and CC5/CC8 genomes, respectively), suggesting that these groups are overrepresented in the database.

WhatsGNU also offers the possibility of reporting the composition of any metadata (e.g., geographical location, disease condition, sequence type (ST), or clonal complex (CC)) associated with exact match alleles in the database. To demonstrate this functionality, we compared a genome of a clinical isolate of *S. aureus* to a CC/ST-curated database. The WhatsGNU report outputs the percentage of genomes from each CC/ST in the database where each allele is seen. As examples, alleles of ArcB and SbnD are shared by most of genomes in CC1/8/398 and CC1/5/8, respectively, while the exact match of TraG is more prevalent, proportionally, outside of CC8 (Fig. 2c, Additional file 4).

To extend the utility of WhatsGNU and to link it to pangenomic analysis, we implemented functions that can use information about the orthology of each allele in each compressed, curated database. If WhatsGNU is given information about orthology of each allele, (e.g., using the clustered_proteins output file from Roary [18]), it can then be used to compare GNU scores of alleles in the same orthologous group, linking panallelomic and pangenomic approaches with known database distributions and frequencies. This functionality can be used to judge whether or not a GNU score is unusual for an orthologous group (see further ortholog variant rarity index in Additional file 1). In Fig. 2, we provide some examples of possible applications of the GNU score for genomic and comparative analysis. One basic approach is to compare GNU scores of alleles that are associated with different biological variables [19]. The heatmap in Fig. 2d shows GNU scores for key components of the TCA cycle, glycolytic pathway, and electron transport chain in 18 different clinical *S. aureus* isolates from atopic dermatitis (AD) and skin and soft tissue infection (SSTI). This type of analysis could uncover differences between groups in user-specified proteins. For instance, there are approximately two times the number of proteins with rare alleles in AD compared to SSTI, perhaps showing novel adaptations in the AD group. Interestingly, high GNU scores show that enolase (Eno) is highly conserved in all isolates, while fumarase (FumC) has more diversity/novelty across genomes, which may signal that FumC is less constrained evolutionarily, and a candidate for possible positive, or relaxed negative, selection. Stark contrasts between GNU scores may signal adaptation or change in function in an individual strain. For instance, SdhA appears to be strongly conserved over the database, and yet isolate number 228_42 has a rare allele with low GNU score.

WhatsGNU can be used for targeted analyses (Fig. 2c, d) and also untargeted (Fig. 2b, e) approaches. Figure 2e and Fig. S3 show volcano plots where two groups of *S. aureus* isolates were compared from patients with AD (*n* = 18) and SSTI (*n* = 49) to find proteins with lower average GNU scores in one group that might represent specific adaptations to the clinical context. This technique uncovered multiple potential genes of interest. For instance, the glutamate dehydrogenase (GudB or GluD) protein that has been implicated in growth in niches where glucose is not as abundant such as SSTI [20], has a lower average GNU score in SSTI isolates compared to AD. Conversely, the MHC class II analog protein, Map, had a lower average GNU score in AD isolates. This gene has a premature stop codon in all of the SSTI isolates and is fully intact in 6 of the AD isolates. A previous study showed that Map is an immunomodulatory protein that may play a role in persistent *S. aureus* infections by reducing activated T cell proliferation [21].

## Conclusion

WhatsGNU leverages natural variation in existing public databases to give context to newly sequenced genomes and protein sequences. The GNU score measures known protein diversity and conservation, identifies the closest matching genomes, and assays for protein novelty. In a matter of seconds on a desktop computer, WhatsGNU will identify completely new sequence variants (GNU score 0), as well as rare protein variants that have been observed only a few times before (low GNU scores). Thus, the GNU score is a convenient way to highlight rare protein variants for targeted functional studies, and to identify possible novel mutations or adaptations.

## Methods

Genomes were downloaded for *S. aureus*, *P. aeruginosa,* and *M. tuberculosis* databases from GenBank [12, 13] using WhatsGNU_get_GenBank_genomes.py, annotated using Prokka [10] and the pangenome was done using Roary [18]. For each species, the proteins of each genome were curated with the strain name, and metadata (CC/ST type) in case of *S. aureus*, and concatenated to one file using WhatsGNU_database_customizer.py. The concatenated file was then used with WhatsGNU_main.py.

Eighty clinical *S. aureus* isolates from an ongoing project (Additional file 5) were used to produce the volcano plot, heatmap, and a single query genome to produce a histogram of GNU scores and to show CC composition using WhatsGNU_plotter.py. A total of 16 isolates from the same project were used to evaluate the effect of sequence quality on GNU = 0 associated error rate. NCTC8325 was used to evaluate the running time of WhatsGNU against blastp [16]. Detailed methods are in Additional file 1.

## Supplementary information

Additional file 1: Supplementary Methods, Supplementary Figures 1, 2 and 3.

Additional file 2: Table S1. Names of the different strains in the five databases. (XLSX 3836 kb)

Additional file 3: Table S2. Names of the different strains used in the collector’s curve in Fig. 1c.

Additional file 4: Example queries and WhatsGNU reports for the 5 databases, WhatsGNU log file, random_sampler script, and input and outputs files for the WhatsGNU and blastp comparison.

Additional file 5: Table S3. Accession numbers for the isolates used in Fig. 2 and Supplementary Figures 1, 2 and 3.

Additional file 6. Review history.

## Authors’ information

Twitter handles: @Ahmed_Microbes (Ahmed M. Moustafa); @pauljplanet (Paul J. Planet).
