# Supplementary material for: WhatsGNU: a tool for identifying proteomic novelty
Source: Genome Biol. 2020 Mar 5;21:58. doi: 10.1186/s13059-020-01965-w (PMC7059281; doi:10.1186/s13059-020-01965-w)
Supplement: Supplementary file 1 — Supplementary Methods, Supplementary Figures 1, 2 and 3. [file 13059_2020_1965_MOESM1_ESM.pdf]

WhatsGNU: Supplementary Material

1 INSTALLATION 2

2 WHATSGNU TOOLBOX 2

3 WHATSGNU USAGE 4

3.1 WHATSGNU_GET_GENBANK_GENOMES.PY 4

3.2 WHATSGNU_DATABASE_CUSTOMIZER.PY 4

3.3 WHATSGNU_MAIN.PY 5

3.4 WHATSGNU_PLOTTER.PY 8

4 AVAILABLE PRECOMPRESSED DATABASES 9

4.1 METHODS FOR ORTHOLOG MODE 9

4.2 METHODS FOR BIG DATA BASIC MODE 11

4.3 DATABASE FORMAT 11

5 METHODS AND RESULTS OF MASH COMPARISON 12

6 METHODS OF FIGURE 1 13

6.1 PANEL C (COLLECTOR'S CURVE) 13

6.2 PANEL D (PERFORMANCE EVALUATION) 13

7 METHODS OF FIGURE 2 14

7.1 PANEL B 14

7.2 PANEL C 14

7.3 PANEL D 15

7.4 PANEL E 15

8 METHODS OF SUPPLEMENTARY FIGURE 1 15

9 METHODS OF SUPPLEMENTARY FIGURE 2 15

10 REFERENCES 16

1 Installation

git clone https://github.com/ahmedmagds/WhatsGNU
cd WhatsGNU/bin
*chmod +x *.py*
pwd
export PATH=$PATH:/path/to/folder/having/WhatsGNU/bin

If it is needed permanently, the last line can be added to .bashrc or .bash_profile.

2 WhatsGNU toolbox

We define **panallelome** as the total set of allelic diversity in a given group of genomes. In this case it is the set of protein alleles that differ by at least one amino acid.

The toolbox is composed of four scripts:

1. WhatsGNU_get_GenBank_genomes.py
   This script downloads genomic fna files or protein faa files from GenBank.
2. WhatsGNU_database_customizer.py
   The protein ids usually have little or no information about the strain so once all proteins are mixed in the database, each strain's information is lost. This script customizes the protein faa files from GenBank, RefSeq, Prokka and RAST by adding a strain name to the start of each protein to later count the top genomes and it also changes the separator to be '|' instead of '_'. This script can also customize the strain names for gff file to be used in Roary for pangenome analysis, if the Ortholog mode is going to be used in WhatsGNU.
3. WhatsGNU_main.py
   In basic mode, this script utilizes the natural variation in public databases to rank protein sequences based on the number of observed exact protein matches (the GNU score) in all known genomes of a particular species. It generates a report for all the proteins in your query in seconds using exact match compression technique. In ortholog mode, the script will additionally link the different alleles of an ortholog group using the clustered proteins output file from Roary or similar pangenome analysis tools. In this mode, WhatsGNU will calculate Ortholog Variant Rarity Index (OVRI) (scale 0-1).

$$OVRI = \frac{\text{Sum of all allele GNU scores in the ortholog group} \leq \text{the GNU score of the allele}}{\text{Sum of GNU scores in the ortholog group}}$$

   We developed this metric to take into consideration the distribution of the GNU scores of other alleles in the same ortholog group. This index represents how unusual a given GNU score is within an ortholog group by measuring how many other protein alleles in the ortholog group have that GNU score or lower. For instance, an allele of GNU=8 in an ortholog group that has 6 alleles with this distribution of GNU scores [300,20,15,8,2,1] will get an OVRI of (8+2+1)/346= 0.03. On the other hand, the allele with GNU=300 will get an OVRI of (300+20+15+8+2+1)/346= 1. An allele with an OVRI of 1 is relatively common regardless of the magnitude of the GNU

score, while an allele with OVRI of 0.03 is relatively rare. This index helps distinguish between ortholog groups with high levels of diversity and ortholog groups that are highly conserved.

Note: The GNU score could be affected by the protein size if the length increases the probability of mutation. In this case a longer protein may obtain lower GNU scores or GNU score of zero more frequently compared to shorter proteins. Similarly, genes that contain homopolymer repeats or other regions that are vulnerable to NGS errors might also end up with lower GNU scores. Truncated proteins would also get a GNU score of zero if they do not appear in the database, and therefore open reading frames at the end of assembled contigs should be validated.

It is important to note also that GNU scores are reflective of the database composition and change as the database grows. GNU scores from well-sampled clades will tend to be higher compared to strains from other under-sampled lineages. However, the change over time is appropriate for the major goal of the GNU score, which is to measure novelty as we learn more about the diversity of genomes in a species.

4. WhatsGNU_plotter.py
   This script plots:
   a. Heatmap of GNU scores of orthologous genes in different isolates.
   b. Metadata distribution bar plot of proteins.
   c. Histogram of the GNU scores of all proteins in a genome.
   d. Volcano plot showing proteins with a lower average GNU score in one group (case) compared to the other (control). The x-axis is the delta average GNU score (Average_GNU_score_case – Average_GNU_score_control) in the ortholog group. Lower average GNU score in cases will have a negative value on the x-axis (red dots) while lower average GNU score in the control group will have positive value on the x-axis (green dots). The y-axis could be drawn as a -log10(*P* value) from Mann–Whitney-Wilcoxon test. In this case, lower average GNU score in one group (upper left for case or upper right for control) would be of interest as shown by a significant *P* value (-log10(*P* value) > 1.3). The y-axis can also be the average OVRI in the case group for negative values on the x-axis or average OVRI in the control group for positive values on the x-axis. In this plot, the regions of interest are:
      i. Lower left: These proteins have lower average GNU scores in the case group compared to control and low OVRI which means the alleles of these proteins in the case group are rarely seen in the entire database of a species.
      ii. Upper left: These proteins have lower average GNU scores in the case group compared to control and high OVRI, which

means the alleles of the proteins in the case group are frequently seen in the entire database of a species.

iii. Lower right: These proteins have lower average GNU scores in the control group compared to case and low OVRI which means the alleles of these proteins in the control group are rarely seen in the entire database of a species.

iv. Upper right: These proteins have lower average GNU score in the control group compared to case and high OVRI which means the alleles of these proteins in the control group are frequently seen in the entire database of a species.

3 WhatsGNU Usage

3.1 WhatsGNU_get_GenBank_genomes.py

3.1.1 Dependency

Python3 [1].

3.1.2 Input

The text file of the GCA numbers of interest.

3.1.3 Command line options

Download proteins faa files:

WhatsGNU_get_GenBank_genomes.py -f GCAs_list.txt Proteomes_folder_name

Download genomic FASTA files:

WhatsGNU_get_GenBank_genomes.py -c GCAs_list.txt Genomes_folder_name

Note: Downloading genomic FASTA files will be needed for MLST typing or if annotation is needed by Prokka [2] to get gff files for pangenome analysis using Roary [3] for the Ortholog mode in WhatsGNU.

3.1.4 Output

A folder of the assembled_genome.fna.gz (-c) or proteome.faa.gz (-f) of the GCAs specified. If -c and -f used together, two output subfolders (contigs and proteins) will be created.

3.2 WhatsGNU_database_customizer.py

3.2.1 Dependency

Python3 [1].

3.2.2 Input

A folder of faa files or gff files to be modified.

3.2.3 Command line options

faa files from GenBank or RefSeq

WhatsGNU_database_customizer.py -c -g -l list.csv prefix_name faa_files_folder_path

faa files from Prokka

WhatsGNU_database_customizer.py -c -p -l list.csv prefix_name faa_files_folder_path

spreadsheet tab-separated text files from RAST

WhatsGNU_database_customizer.py -c -r -l list.csv prefix_name RAST_spreadsheets_folder_path

gff files from Prokka

WhatsGNU_database_customizer.py -i -s -l list.csv prefix_name gff_files_folder_path

Note: In its basic mode, the customizer script uses the file name as a locustag. The script can also accept a list.csv (comma-separated) of 3+ columns: file_name, old locustag, new locustag and optionally metadata. In the latter case, if metadata are provided, the script will concatenate the new locustag with metadata using '_' as a separator. The new locustag in this case will be: new_locustag_metadata_. In case of GenBank, RefSeq and RAST, use NA for the old locustag column in the list.csv file. The -c option will provide one concatenated file of all input files, which is recommended for protein faa files while the -i option will return individual files, which is required for gff files.

3.2.4 Output

A prefix_concatenated.faa, individual modified faa files or individual modified gff files. If metadata were provided in the list.csv with the -l option, the script will output a metadata_frequency.csv file that has the metadata frequencies in the database.

3.3 WhatsGNU_main.py

3.3.1 Dependency

- Python3 [1].
- Blastp [4] (optional).

3.3.2 Input

General

1. Database (precompressed (.pickle, .txt or .db) with -d or raw (prefix_concatenated.faa or folder of multiple faa files)) with -m.
2. Query protein FASTA file (.faa) or folder of multiple query files.

Optional

1. Clustered_proteins output file from roary with -m option.
2. The CSV file of metadata frequencies in the database.

3.3.3 Command line options

Use your own database

First time use with unprocessed database in basic mode

WhatsGNU_main.py -m prefix_concatenated.faa -a query.faa

First time use with unprocessed database in Ortholog mode

WhatsGNU_main.py -m prefix_concatenated.faa -a -r clustered_proteins query.faa

Assign prefix for output compressed db using -p (Default: WhatsGNU_compressed_database)

WhatsGNU_main.py -m prefix_concatenated.faa -a -p compressed_db query.faa

Note: -a produces compressed_db.pickle in addition to .txt for faster subsequent runs. There is -j option which produces sql.db for computers with limited memory option.

Subsequent uses with a precompressed database

WhatsGNU_main.py -d compressed_db.pickle -dm basic query.faa

or

WhatsGNU_main.py -d compressed_db.pickle -dm ortholog query.faa

for folder of multiple (.faa) query files

WhatsGNU_main.py -d compressed_db.pickle -dm basic [or ortholog] folder_faa/

Use more features

Assign output folder name using -o (Default: WhatsGNU_results_timestamp).

WhatsGNU_main.py -d compressed_db.pickle -dm ortholog -o output_results_folder query.faa

Create a file of each protein with all associated ids from the database.

WhatsGNU_main.py -d compressed_db.pickle -dm ortholog -i -o output_results_folder query.faa

Create a file of top 10 genomes with hits.

WhatsGNU_main.py -d compressed_db.pickle -dm ortholog -t query.faa

Note: The top 10 closest genomes to a new genome in the database are the genomes with the highest number of exact matches.

Check how many hits you get from a particular genome in the database.

WhatsGNU_main.py -d compressed_db.pickle -dm ortholog -t -s strain_name query.faa

Get metadata composition of your hits in the report.

WhatsGNU_main.py -d compressed_db.pickle -dm ortholog -e metadata_frequencies.*csv query.faa*

Get a fasta (.faa) file of all proteins with GNU score of zero.

WhatsGNU_main.py -d compressed_db.pickle -dm ortholog -f query.faa

Note: -dm basic will work for all the previous commands.

The following options work with -dm ortholog:

Run blastp on the proteins with GNU score of zero and modify the report with ortholog information.

WhatsGNU_main.py -d compressed_db.pickle -dm ortholog -b query.faa

Note: blastp has been used in concordance with the recent literature describing a common misconception regarding the BLAST parameter -max_target_seqs [5, 6].

Get the output report of blastp run (works with -b).

WhatsGNU_main.py -d compressed_db.pickle -dm ortholog -b -op query.faa

Select a blastp percent identity cutoff value [Default 80], range(0,100).

WhatsGNU_main.py -d compressed_db.pickle -dm ortholog -b –w 95 query.faa

Select a blastp percent coverage cutoff value [Default 80], range(0,100).

WhatsGNU_main.py -d compressed_db.pickle -dm ortholog -b –c 40 query.faa

Select an OVRI cutoff value [Default 0.045], range (0-1).

WhatsGNU_main.py -d compressed_db.pickle -dm ortholog -ri 0.09 *query.faa*

Use all features together

WhatsGNU_main.py -d compressed_db.pickle -dm ortholog
-o output_results_folder -i -t -s strain_name -e metadata_frequencies.csv -f -b
-op –w 95 –c 40 -ri 0.09 query.faa

3.3.4 Output

Always with -m or -d

query_WhatsGNU_report.txt

Note: If -e option is used, metadata percentages' columns will be added to the report.

WhatsGNU_date_time.log

Always with -m

compressed_db.txt (if -a, compressed_db.pickle will be created)
*compressed_db*_orthologs.faa (if “-r clustered_proteins” is used with -m)
*compressed_db*_orthologs_info.txt (if “-r clustered_proteins” is used with -m)

Note: If -b is used, WhatsGNU will search for *compressed_db*_orthologs.faa and *compressed_db*_orthologs_info.txt in the same path for the compressed database as they are needed for the blastp run.

Optional

query_WhatsGNU_hits.txt (-i, each protein with all hits_ids from the database)
query_WhatsGNU_topgenomes.txt (-t, top 10 genomes with hits to your query)
query_WhatsGNU_zeros.faa (-f, a fasta (.faa) file of all proteins with GNU score of zero)
query_WhatsGNU_zeros_blast_report.txt (-op, output report of blastp run)

3.4 WhatsGNU_plotter.py

3.4.1 Dependency

- Python3 [1].
- Blastp [4].
- NumPy [7, 8].
- Matplotlib [9].
- SciPy [10].

3.4.2 Input

A folder of query_WhatsGNU_report.txt files.

3.4.3 Command line options

Heatmap

Plot a heatmap of GNU scores for these proteins in proteins.faa using this strains' order. Assign a title using -t. Font size and figure size (w,h) are given by -f and -fs, respectively. Annotate the heatmap cells with OVRI rare tag using -r option.

WhatsGNU_plotter.py -hp ortholog -q proteins*.faa -d strains_order.txt -t title -r -f 14 -fs 14 10 prefix_name WhatsGNU_reports_folder/*

Metadata percentage distribution

Plot a metadata percentage bar plot for the GNU scores of the proteins in proteins.faa for each WhatsGNU report.

WhatsGNU_plotter.py -mb basic -q proteins*.faa prefix_name WhatsGNU_reports_folder/*

Histogram

Plot a blue histogram of the GNU scores for each WhatsGNU report using 100 bins and get a text file showing novel and conserved proteins with -p option to assign cutoffs.

WhatsGNU_plotter.py -x -e blue -b 100 -p 50 5000 prefix_name WhatsGNU_reports_folder/

Volcano plot

Plot two scatterplots that shows either statistical significance (*P* value) or average OVRI versus magnitude of change (Delta_average_GNU_Score). The case/control tag is provided in isolates_case_control_tag.csv. The option -c 100 is a percentage of isolates a protein must be in to be included. A summary statistics file is also created.

WhatsGNU_plotter.py -st isolates_case_control_tag.csv -c 100 prefix_name WhatsGNU_reports_folder/

Use many features together

WhatsGNU_plotter.py -hp ortholog -q proteins*.faa -d strains_order.txt -t title -r -f 16 -fs 14 10 -mb ortholog -x -e blue -b 100 -st isolates_case_control_tag.csv -c 100 prefix_name WhatsGNU_reports_folder/*

3.4.4 Output

A heatmap, metadata percentage distribution bar plot, histogram and two volcano plots and summary statistics files.

4 Available Precompressed Databases

Ortholog Mode:

1. *Staphylococcus aureus* Ortholog. Version: 06/14/2019 (compressed 27,213,667 proteins in 10350 genomes to 571,848 protein variants) [11].
2. *Mycobacterium tuberculosis* Ortholog. Version: 07/09/2019 (compressed 26,794,006 proteins in 6563 genomes to 434,725 protein variants) [11].
3. *Pseudomonas aeruginosa* Ortholog. Version: 07/06/2019 (compressed 14,475,742 proteins in 4712 genomes to 1,288,892 protein variants) [11].

Big Data basic Mode:

1. *Salmonella enterica* Basic. Version: 08/29/2019 (compressed 975,262,506 proteins in 216,642 genomes to 5,056,335 protein variants) [11].
2. Staphopia (*Staphylococcus aureus*) Database Basic. Version: 06/27/2019 (compressed 115,178,200 proteins in 43,914 genomes to 2,228,761 protein variants) [11].

These 4 species are in the top 10 species with assembled genomes on GenBank [12], and are categorized as critical and high priority pathogens in the global priority pathogens list [13].

4.1 Methods for Ortholog Mode

4.1.1 The genome assemblies for the species were downloaded from NCBI.

1. *S. aureus* [14].
2. *M. tuberculosis* [15].
3. *P. aeruginosa* [16].

There were 10374, 6582 and 4804 genomes for *S. aureus* (06/14/2019), *M. tuberculosis* (07/09/2019) and *P. aeruginosa* (07/06/2019) on GenBank [17, 18], respectively. We excluded 24, 19 and 92 contaminated or misassembled genomes for *S. aureus*, *M. tuberculosis* and *P. aeruginosa*, respectively, as per the note in the assembly column of the NCBI genome assembly and annotation report [14-16]. The genomes were downloaded using the WhatsGNU_get_GenBank_genomes.py script as following:

```
WhatsGNU_get_GenBank_genomes.py -c Sau_database_061419_GCAs.txt Sau_database_061419
WhatsGNU_get_GenBank_genomes.py -c TB_database_070919_GCAs.txt TB_database_070919
WhatsGNU_get_GenBank_genomes.py -c Pa_database_070619_GCAs.txt Pa_database_070619
```

The assembled genomes were then annotated using Prokka as following:

```
for i in `cat Sau_prokka_file_names.list`;do prokka --kingdom Bacteria --outdir prokka_Sau/prokka_$i --gcode 11 --genus Staphylococcus --species aureus --strain $i --prefix $i --locustag $i Sau_database_061419/$i*.fna; done
```

for i in `cat TB_prokka_file_names.list`;do prokka --kingdom Bacteria --outdir prokka_TB/prokka_$i --gcode 11 --genus Mycobacterium --species tuberculosis --strain $i --prefix $i --locustag $i TB_database_070919/$i.fna; done*
for i in `cat Pa_prokka_file_names.list`;do prokka --kingdom Bacteria --outdir prokka_Pa/prokka_$i --gcode 11 --genus Pseudomonas --species aeruginosa --strain $i --prefix $i --locustag $i Pa_database_070619/$i.fna; done*

4.1.2 For *M. tuberculosis* and *P. aeruginosa*, the strain name and GCA number were concatenated in excel to look strain_GCA_number. A csv file was then created to have the file_name.gff[or .faa],old_locustag,new_locustag. The WhatsGNU_database_customizer.py was then used to modify the faa and gff files as following:

WhatsGNU_database_customizer.py -i -s -l TB_names_list_gff.csv TB_modified_gff TB_gff/
WhatsGNU_database_customizer.py -c -p -l TB_names_list_faa.csv TB_modified_faa TB_faa/
WhatsGNU_database_customizer.py -i -s -l Pa_names_list_gff.csv Pa_modified_gff Pa_gff/
WhatsGNU_database_customizer.py -c -p -l Pa_names_list_faa.csv Pa_modified_faa Pa_faa/

4.1.3 For *S. aureus*, the mlst tool (https://github.com/tseemann/mlst) was used to type each strain and the ST type or CC type were added to the strain name and GCA number as metadata. A csv file was created to have the file_name.gff[or .faa],old_locustag,new_locustag,CC[or ST]number. The WhatsGNU_database_customizer.py was then used to modify the locustag in faa and gff files to be strain_GCA_number_CC[or ST]number_ as follows:

WhatsGNU_database_customizer.py -i -s -l Sau_names_list_gff.csv Sau_modified_gff Sau_gff/
WhatsGNU_database_customizer.py -c -p -l Sau_names_list_faa.csv Sau_modified_faa Sau_faa/

4.1.4 Pangenome analysis for each species was done using Roary [3] as follows:

roary -i 80 Sau_modified_gff/.gff*
roary -i 80 TB_modified_gff/.gff*
roary -i 80 Pa_modified_gff/.gff*

4.1.5 The database_concatenated.faa was then used with WhatsGNU with the database compression option (-m) and the reference genome of each species as query as follows:

time WhatsGNU_main.py -m Sau_modified_faa_concatenated.faa -r clustered_proteins -a -o Sau_Ortholog_10350 -p Sau_Ortholog_10350 NCTC8325.faa

or

time WhatsGNU_main.py -m TB_modified_faa_concatenated.faa -r clustered_proteins -a -o TB_Ortholog_6563 -p TB_Ortholog_6563 H37Rv.faa

or

time WhatsGNU_main.py -m Pa_modified_faa_concatenated.faa -r clustered_proteins -a -o Pa_Ortholog_4712 -p Pa_Ortholog_4712 PAO1.faa

4.2 Methods for Big Data Basic Mode

The 200K and 43,914 Prokka-annotated genomes of *S. enterica* from Enterobase [19] and *S. aureus* from Staphopia [20], respectively, were compressed using WhatsGNU as follows:

time WhatsGNU_main.py -m Salmonella_database.faa CT18.faa

or

time WhatsGNU_main.py -m Sau_Staphopia_database.faa NCTC8325.faa

The names of the different strains in the databases are listed in **Additional file 2**. These 4 query files (H37Rv.faa, PAO1.faa, NCTC8325.faa and CT18.faa), their 5 WhatsGNU reports, and WhatsGNU_date_time.log are available in **Additional file 4**.

4.3 Database format

Using WhatsGNU with –m option compresses the database in two formats for convenience; a database.txt format that will need shorter time for subsequent uses. It also uses the pickle module [21] of Python3 [1] to save it as a database.pickle. The five databases are available to download [11] and also by visiting the link or using the wget command as follows:

Sau_Ortholog

wget -O Sau.zip
https://www.dropbox.com/sh/p292mia4oc99hx6/AACPuv7uoYUkZ1WCBDX0XPSVa?dl=0
unzip Sau.zip -d WhatsGNU_Sau_Ortholog

TB_Ortholog

wget -O TB.zip
https://www.dropbox.com/sh/8nqowtd4fcf7dgs/AAAdXiqcxTsEqflAyNE9TWwRa?dl=0
unzip TB.zip -d WhatsGNU_TB_Ortholog

Pa_Ortholog

wget -O Pa.zip
https://www.dropbox.com/sh/r0wvoig3alsz7xg/AABPoNu6FdN7zG2PP9BFezQYa?dl=0
unzip Pa.zip -d WhatsGNU_Pa_Ortholog

Staphopia

wget -O Sau_Staphopia_basic_43914.pickle
https://www.dropbox.com/s/bcs922768tjrwwg/Sau_Staphopia_basic_43914.pickle?dl=0

Salmonella Enterobase

wget -O Senterica_Enterobase_basic_216642.pickle
https://www.dropbox.com/s/gbjengikpynxo12/Senterica_Enterobase_basic_216642.pickle?dl=0

Note: The Senterica_Enterobase_basic_216642.pickle database needs a 85 GB of RAM to load.

5 Methods and Results of Mash comparison

We compared the WhatsGNU -t option with the Mash sketch/dist functions [17] using the *S. aureus* genome NCTC8325.
The Mash tool [22] was used as follows:

time mash sketch NCTC8325.fna
time mash dist refseq.genomes.k21s1000.msh NCTC8325.fna.msh > dist.tab
time sort -gk3 dist.tab | head

WhatsGNU was used as follows:

time WhatsGNU_main.py -d Sau_Ortholog_10350.pickle -dm ortholog -t NCTC8325.faa

The time needed for Mash and WhatsGNU to finish was 25 and 23 seconds, respectively, on a MacBook Pro desktop computer with a single processor (3.3 GHz Intel Core i7) and 16 GB of RAM. It is worth noting that roughly 13 seconds were used to load the database so the time would be reduced to find top genomes for multiple isolates to be only 10 seconds per genome while for Mash 25 seconds are needed to sketch each genome.

Excluding a second copy of NCTC8325 (GCA_900475245.1) in the GenBank database compared to RefSeq, the top 10 genomes from WhatsGNU were as follows:

NCTC_8325_GCA_000013425.1_CC8_	2283 (hit 1 in Mash)
FDAARGOS_10_GCA_001018725.2_CC8_	2263 (hit 6 in Mash)
HG001_GCA_001900185.1_CC8_	2253 (hit 2 in Mash)
FDAARGOS_13_GCA_001019335.2_CC8_	2243 (hit 4 in Mash)
HG003_GCA_000736455.1_CC8_	2233 (hit 3 in Mash)
21189_GCA_000205345.2_CC8_	2194 (hit 5 in Mash)
FDAARGOS_23_GCA_001018885.2_CC8_	2160 (hit 7 in Mash)
FDAARGOS_28_GCA_001018955.2_CC8_	2159 (hit 8 in Mash)
FDAARGOS_31_GCA_001019015.2_CC8_	2147 (hit 9 in Mash)
NRS133_GCA_001018915.2_CC8_	2125 (hit 10 in Mash)

Note: WhatsGNU additionally provides the strain name and metadata (clonal complexes) details.

6 Methods of Figure 1

6.1 Panel C (Collector's curve)

A collector's curve was used to compare the size of the panallelome of available genomes of *S. aureus* on GenBank [17, 18] and Staphopia [20] which would reflect any biases in sampling for both databases. A collector's curve in this case expresses the number of unique alleles as a function of the number of genomes sequenced. The genomes available on GenBank [17, 18] at two different time points (8524 and 10350) were used with WhatsGNU and the panallelome size was recorded. A script (random_sampler.py in **Additional file 4**) was then used to randomly select 1000, 2000, 4000, 8524,10350, 20000 and 30000 genomes from the 43914 *S. aureus* genomes available on Staphopia [20]. The random sampling step was done three independent times with replacement and was run with WhatsGNU. Panallelome size was recorded and plotted in GraphPad Prism v.7.

random_sampler.py -n 20000 random_sample_folder_name Sau_staphopia/

Note: -n was changed to randomly select 1000, 2000, 4000, 8524,10350 20000 and 30000 with replacement. The curve showed that genomes in Staphopia [20] had greater sampling breadth compared to GenBank [17, 18], which was reflected by the shift in the number of alleles that were obtained at the two data points 8524 and 10350. The names of the different strains used in the collector's curve are listed in **Additional file 3**.

6.2 Panel D (Performance evaluation)

A MacBook Pro desktop computer with a single processor (3.3 GHz Intel Core i7) and 16 GB of RAM was used for the evaluation.

6.2.1 WhatsGNU

One file and two folders of 100 and 1000 copies of *S. aureus* NCTC 8325 were used as a query for WhatsGNU in three different runs and wall running times were noted in seconds.

time WhatsGNU_main.py -d Sau_012119.pickle -dm basic NCTC8325.faa
time WhatsGNU_main.py -d Sau_012119.pickle -dm basic folder_100/
time WhatsGNU_main.py -d Sau_012119.pickle -dm basic folder_1000/

6.2.2 blastp

To reduce computational costs for blastp, we multiplied the wall running time of one copy of *S. aureus* genome NCTC 8325 by 100 and 1000, respectively.

makeblastdb -in Sau_012119.faa -input_type fasta -dbtype prot -max_file_sz 2000000000B
time blastp -query NCTC8325.faa -db Sau_012119.faa -qcov_hsp_perc 100 -max_target_seqs 8500 -outfmt "7 sacc evalue qcovs pident" -out NCTC8325_blast.results

To compare the output of WhatsGNU with blastp as a gold standard, we used a concatenated file of a small dataset of 3 strains' protein FASTA files as either a blastp subject or WhatsGNU input and two proteins (50S ribosomal protein L9 and DnaA) from NCTC8325 as queries as follows:

blastp -query NCTC8325_2_ptn.faa -subject blastp_db.faa -evalue 1e-104 -outfmt '6 qseqid sacc evalue qcovs pident' -out NCTC8325_2ptn_blast_report.txt
WhatsGNU_main.py -m blastp_db.faa NCTC8325_2_ptn.faa

The number of exact matches were counted in the blast report and compared with WhatsGNU results. The number of the exact matches reported by blastp and WhatsGNU (GNU score) were identical. The query and subject files and the two output reports NCTC8325_2ptn_blast_report.txt and NCTC8325_2_ptn_WhatsGNU_report.txt are available in **Additional file 4**.

7 Methods of Figure 2

7.1 Panel B

The GNU scores of all the proteins in a SSTI *S. aureus* clinical-CC8-USA300 isolate "*SSTI_179_1*" were used to produce a histogram (panel B). It is important to note that genomes from different lineages will have different histograms based on the composition of the database.

WhatsGNU_plotter.py -x -e blue -b 103 -p 100 10000 SSTI_179_1_histogram WhatsGNU_reports_folder/

Note: WhatsGNU_reports_folder/ has SSTI_179_1_WhatsGNU_report.txt

Note: There were 37 and 170 proteins with GNU < 100 and > 10,000 representing the novelty and ultra-conserved peaks, respectively (in **Additional file 4**). The novel peak had some virulence proteins such as Staphostatin B and Clumping factor A, transcriptional regulators and IS elements. On the other hand, the ultra-conserved peak had proteins like GpsB (Cell cycle protein), DivIC (Cell division protein), multiple 30S and 50S ribosomal proteins, SigA (RNA polymerase sigma factor) and ATP synthase subunits. In supplementary figure 2 we annotated the proteins in GNU score bin category with gene ontology terms. This GO-annotated histogram showed that some accessory proteins annotated with biological processes such as adhesion and aggregation are located in the novelty peak with low GNU scores while proteins involved in cellular processes and cellular component organization or biogenesis tend to be more conserved.

7.2 Panel C

A SSTI *S. aureus* clinical-CC8-USA300 isolate "*SSTI_179_1*" was used to create a report with CC/ST composition using –c option. The percentages for CC 5, 8, 22, 398,30 and 1 for GNU scores of three proteins, SbnD (staphyloferrin B export MFS transporter), TraG (Transfer complex protein) and ArcB (ornithine carbamoyltransferase) were plotted as bar graphs (panel C) using the metadata_barplot option in the WhatsGNU_plotter script.

WhatsGNU_plotter.py -mb ortholog -s CC_included.txt -q SbnD_TraG_ArcB.faa SSTI_179_1_metadata WhatsGNU_reports_folder/

Note: WhatsGNU_reports_folder/ has SSTI_179_1_WhatsGNU_report.txt (in **Additional file 4)**

7.3 Panel D

Eighteen paired clinical isolates of *S. aureus* from atopic dermatitis (AD) and soft and skin tissue infection (SSTI) representing different clonal complexes from the same ongoing project were used with the WhatsGNU ortholog mode and GNU scores for key components of the TCA cycle, the glycolytic pathway, and terminal components of the electron transport chain were used to produce a heatmap.

WhatsGNU_plotter.py -hp ortholog -q proteins.faa -r -d strains_order.txt -f 14 -fs 14 10 AD_SSTI_heatmap SSTI_AD_paired_GNU_reports/

7.4 Panel E

Two volcano plots showing proteins with a lower average GNU score in one group of 18 CC8 *S. aureus* isolates from atopic dermatitis cases compared to another group of 49 CC8 *S. aureus* isolates from soft and skin tissue infection cases and vice versa. The accession numbers for the 67 isolates are provided in **Additional file 5.** The volcano plot showing the difference in average GNU scores in each group against average OVRI is in supplementary figure 3.

WhatsGNU_main.py -d Sau_Ortholog_10350.pickle -b -op -f -o AD_SSTI_GNU_reports -dm ortholog AD_SSTI_faa/

WhatsGNU_plotter.py -st AD_SSTI_case_control.csv -cc AD SSTI AD_SSTI_Volcano AD_SSTI_GNU_reports/

8 Methods of Supplementary Figure 1

A total of 16 *S. aureus* clinical isolates from the same ongoing project were used to evaluate the effect of sequence quality on GNU score of zero (error rate). The 16 isolates were run against the *S. aureus* database. GNU scores of zero and contig numbers were used to produce a linear regression graph in GraphPad Prism v.7.

WhatsGNU_main.py -d Sau_Ortholog_10350.pickle -f -dm ortholog 16_error_rate_*faa/*

9 Methods of Supplementary Figure 2

The proteome of the SSTI *S. aureus* clinical-CC8-USA300 isolate "*SSTI_179_1*" was blasted against NCTC 8325 and 80% identity and 80% coverage were used to call an ortholog from NCTC 8325. The 2475 mapped protein IDs from NCTC 8325 were then used in GOQuick [23, 24] to annotate the proteins with biological processes gene ontology terms. A total of 1535 proteins were annotated. The GO annotations were limited to 22 biological processes (metabolic process, cellular process, localization, biological regulation, response to stimulus, cellular component organization or biogenesis, multi-organism process, signaling, detoxification, cell killing, biological adhesion, reproduction, developmental process, nitrogen utilization, cell aggregation, carbon utilization, carbohydrate utilization, growth, locomotion, sulfur utilization, phosphorus utilization and pigmentation). There were 1145 proteins that were not annotated and were reported as unclassified. The GNU scores of all the proteins were used to produce a histogram with a y-axis showing the composition of biological process hits in GOQuick for each of the 103 GNU score bins.

```
blastp -query SSTI_179_1.faa -subject uniprot-proteome_UP000008816.fasta -max_target_seqs 5 -max_hsps 1 -outfmt '6 qseqid sseqid evalue qcovs pident' -out SSTI_179_1_blast_report.txt
```

The FASTA files and the WhatsGNU reports as inputs and outputs, respectively, that were used to produce panels in Figures 1 and 2 and supplementary figures 1, 2 and 3 are available to download [11].

10 References

1. **Python3: https://www.python.org/. Accessed 05 February 2019.**
2. Seemann T: **Prokka: rapid prokaryotic genome annotation.** *Bioinformatics* 2014.
3. Page AJ, Cummins CA, Hunt M, Wong VK, Reuter S, Holden MT, Fookes M, Falush D, Keane JA, Parkhill J: **Roary: rapid large-scale prokaryote pan genome analysis.** *Bioinformatics* 2015, **31:**3691-3693.
4. Camacho C, Coulouris G, Avagyan V, Ma N, Papadopoulos J, Bealer K, Madden TL: **BLAST+: architecture and applications.** *BMC Bioinformatics* 2009, **10:**421.
5. Shah N, Nute MG, Warnow T, Pop M: **Misunderstood parameter of NCBI BLAST impacts the correctness of bioinformatics workflows.** *Bioinformatics* 2019, **35:**1613-1614.
6. Madden TL, Busby B, Ye J: **Reply to the paper: Misunderstood parameters of NCBI BLAST impacts the correctness of bioinformatics workflows.** *Bioinformatics* 2019, **35:**2699-2700.
7. Walt Svd, Colbert SC, Varoquaux G: **The NumPy Array: A Structure for Efficient Numerical Computation.** *Computing in Science & Engineering* 2011, **13:**22-30.
8. Oliphant TE: *Guide to NumPy.* Trelgol Publishing; 2006.
9. Hunter JD: **Matplotlib: A 2D Graphics Environment.** *Computing in Science & Engineering* 2007, **9:**90-95.
10. Jones E, Oliphant T, Peterson P, Others: **SciPy: Open Source Scientific Tools for Python (http://www.scipy.org).** 2001.
11. Moustafa AM, Planet PJ: **Supplemental Datasets for: WhatsGNU: A Tool For Identifying Proteomic Novelty.** Zenodo. http://doi.org/10.5281/zenodo.3633425 (2020).
12. **NCBI GenBank assembly database: https://www.ncbi.nlm.nih.gov/assembly/. Accessed 03 February 2020.**
13. **WHO Priority Pathogens List (September 2017): https://www.who.int/medicines/areas/rational_use/prioritization-of-pathogens/en/. Accessed 04 February 2019.**
14. **Genome Assembly and Annotation report for *Staphylococcus aureus*: https://www.ncbi.nlm.nih.gov/genome/genomes/154?. Accessed 14 June 2019.**
15. **Genome Assembly and Annotation report for *Mycobacterium tuberculosis*: https://www.ncbi.nlm.nih.gov/genome/genomes/166?. Accessed 09 July 2019.**
16. **Genome Assembly and Annotation report for *Pseudomonas aeruginosa*: https://www.ncbi.nlm.nih.gov/genome/genomes/187?. Accessed 06 July 2019.**

17. Sayers EW, Cavanaugh M, Clark K, Ostell J, Pruitt KD, Karsch-Mizrachi I: **GenBank.** *Nucleic Acids Res* 2019, **47:**D94-D99.
18. **GenBank Database: ftp://ftp.ncbi.nlm.nih.gov/genomes/genbank/bacteria/. Accessed 26 August 2019**
19. Alikhan NF, Zhou Z, Sergeant MJ, Achtman M: **A genomic overview of the population structure of Salmonella.** *PLoS Genet* 2018, **14:**e1007261.
20. Petit RA, 3rd, Read TD: **Staphylococcus aureus viewed from the perspective of 40,000+ genomes.** *PeerJ* 2018, **6:**e5261.
21. **Pickle Module: https://docs.python.org/3/library/pickle.html. Accessed 05 February 2019.**
22. Ondov BD, Treangen TJ, Melsted P, Mallonee AB, Bergman NH, Koren S, Phillippy AM: **Mash: fast genome and metagenome distance estimation using MinHash.** *Genome Biol* 2016, **17:**132.
23. Huntley RP, Sawford T, Mutowo-Meullenet P, Shypitsyna A, Bonilla C, Martin MJ, O'Donovan C: **The GOA database: gene Ontology annotation updates for 2015.** *Nucleic Acids Res* 2015, **43:**D1057-1063.
24. Binns D, Dimmer E, Huntley R, Barrell D, O'Donovan C, Apweiler R: **QuickGO: a web-based tool for Gene Ontology searching.** *Bioinformatics* 2009, **25:**3045-3046.

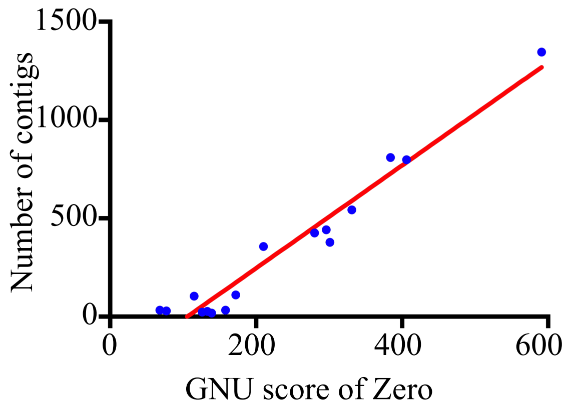

Figure S1. Relationship between sequence assembly quality and GNU score of Zero. More proteins with GNU scores of zero (representing potential errors) are seen as number of contigs increases, and sequence quality decreases

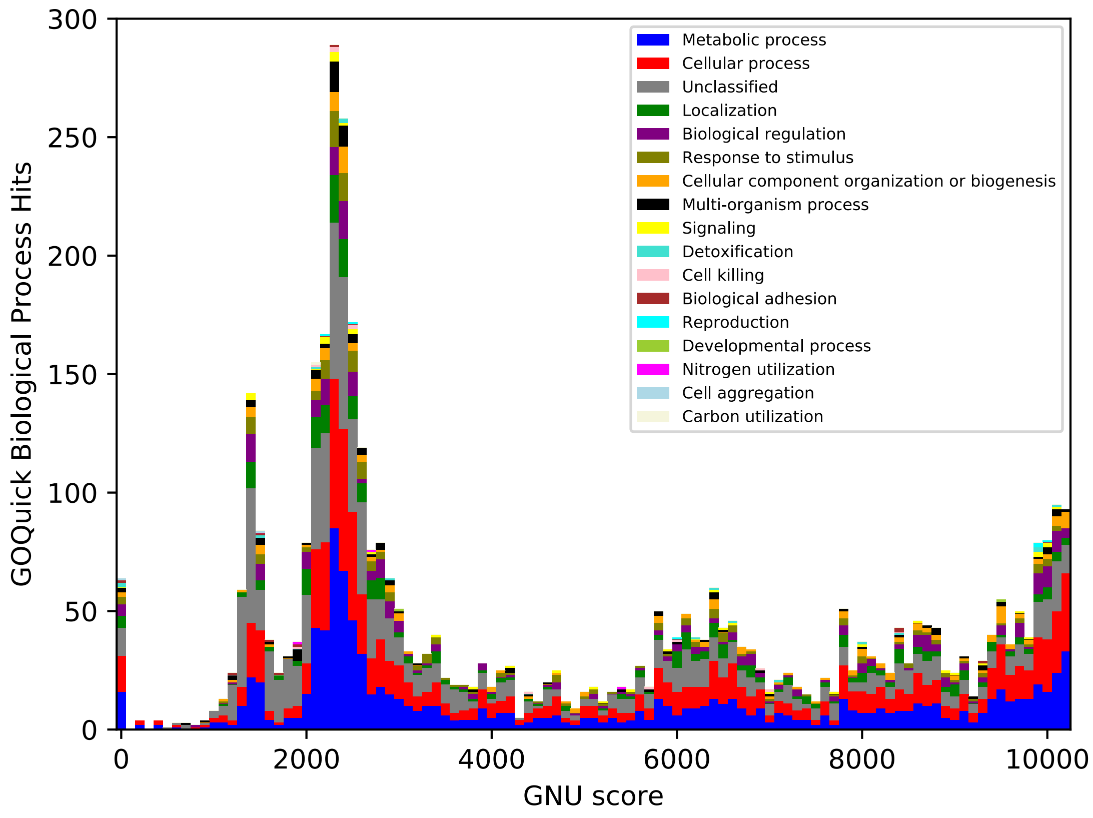

Figure S2. A histogram of GNU scores of a clinical-CC8-USA300 *S. aureus* genome “SSTI_179_1” with gene ontology biological processes’ terms annotations

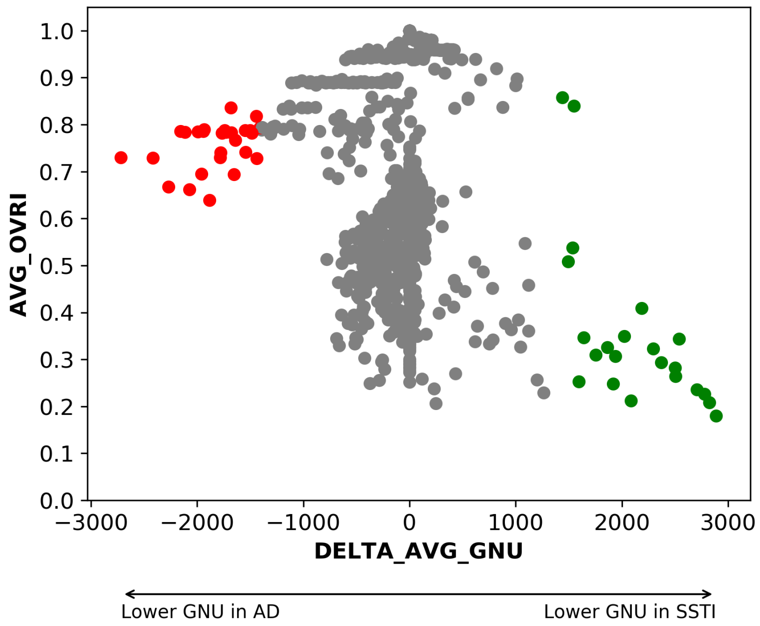

Figure S3. Volcano plot showing proteins with a lower average GNU score in a case group (atopic dermatitis) compared to a control group (soft and skin tissue infection). Proteins with lower average GNU score in the AD case group of 18 CC8 *S. aureus* isolates are shown in red. Proteins with lower average GNU score in the SSTI control group of 49 CC8 *S. aureus* isolates are shown in green. The y-axis is the average Ortholog Variant Rarity index (OVRI).
