# Supplementary material for: WhatsGNU: a tool for identifying proteomic novelty
Source: Genome Biol. 2020 Mar 5;21:58. doi: 10.1186/s13059-020-01965-w (PMC7059281; doi:10.1186/s13059-020-01965-w)
Supplement: Supplementary file 6 — Review history. [file 13059_2020_1965_MOESM6_ESM.docx]

**First round**

**Reviewer 1**

WhatsGNU is a compression software designed to rapidly reduce genbank formatted proteomes to libraries of exact matches. Individual proteomes can be queried for the number of identical matches and hence a "novelty score" can be calculated. The results section is a series of vignettes of potential uses of the software, using moderately sized databases of S. aureus, M. tuberculosis and P. aeruginosa as examples. No novel scientific conclusions were drawn about the organisms as a result of these analyses.

In my hands the software installed easily and worked as advertised (see below). It was impressively fast - better performance than cd-hit, a software with similar applications. Downsides were the lack of natural support for custom databases and no built in function to generate summary tables and publication quality plots.

Overall, I found this to be an interesting software but without a really great application. The major strength is allowing users a way to quickly get an idea of the proteome composition of a set of genomes from genbank. However, after doing this there is not much more that can be done with the WhatsGNU database. This is because of the principal limitation of the software - it can only return 100% matches, which means the database isn't a true pangenome representation and you would need to go on and run a software like ROARY on your data anyway. Other software exists to provide a "quick peek", such as mash (albeit with a completely different approach) but mash sketches can also be used for other applications such as calculating average nucleotide identity, building quick trees etc.

Minor/Specific Points

This software should work on gene sequences as well as protein. Surprised that the authors did not mention this, as its a potential alternative for cgMLST.

Lline 69 - Need to spell out what are different challenges on human versus bacterial proteomes. WhatsGNU itself is not limited to bacteria and could be used on human datasets.

Line 81 - would be impressive to showcase WhatsGNU on the truly large datasets out there now - e.g 200,000+ Salmonella genomes.

Line 109 - I think here you need to compare the strengths of WhatsGNU with mash, which is the tool most commonly used to answer this particular question at the moment.

Naming and Visibility:

I realize it is late in the game, but I think it might be worthwhile to reconsider the naming of "WhatsGNU". The naming is fun, but I think the usage of "GNU" is likely to confuse users. Is this tool a part of (or going to be) the GNU Project? If not, since GNU is a popular operating system and has many tools under its umbrella, I'm afraid WhatsGNU will never escape a false association with GNU. A Google search of GNU returns 100+ million results.

Also, a quick Google search of "What's GNU" returns the board game "What's GNU": https://www.amazon.com/Think-Fun-7760-Whats-Gnu/dp/B00073JTQQ

As well as general posts regard what GNU is and what the GNU Project is.

I was unsuccessful finding the WhatsGNU GitHub repo through Google search using terms like:

whatsgnu bioinformatics

whatsgnu github

whatsgnu gene novelty unit github

gene novelty unit github

But I am unsure how long the repo has been public. It may not have been indexed yet. Although, anything with "gnu" in the search was littered with results associated with GNU.

I did find this repo, https://github.com/mioux/whats-gnu, but it has been untouched for at least 4 years.

Software remarks

*I followed installation information at https://github.com/ahmedmagds/WhatsGNU

Git clone, chmod 755, add to path all make sense and are pretty standard. I created a custom database based on 59 complete bacterial genomes. Everything completed quickly (~2seconds). A total of 100,960 proteins was compressed to 35,565 unique proteins. I received the expected report for the query. I created another database which included the 959 draft assemblies. This compressed 1,700,211 proteins into a database of 181,465 unique proteins in ~20 seconds. I then processed to query the 59 completed genomes against this new 959 sample database. Everything seemed to have finished successfully. I included "--topgenomes", "--hits", and "--strainhits" in the execution. The "hits" files were created, the "topgenomes" and "strainhits" does not work for custom data. As "topgenomes" and "strainhits" is currently written it only works for strains with "_GCA_" in their name. I looked into the code, and it looks like "tophits" and "strainhits" will only work for GenBank assemblies (GCA) and not RefSeq assemblies (GCF) or custom assembly names. The authors acknowledge this problem but their solution is clumsy (attaching "GCA" to protein descriptions, and beyond the reach of many/most users). Currently this limits the functionality of WhatsGNU.

* One thing to consider is if you call "WhatsGNU.py" without arguments it exits with a status 2. Makes sense since argparse recognizes `query_faa` was missing. A better approach might be to print the usage and exit with status code 0 if no arguments are given. Something like:

```

if len(sys.argv) == 1:

PARSER.print_help()

sys.exit(0)

ARGS = PARSER.parse_args()

```

* Are there plans to submit this to Bioconda (https://bioconda.github.io/)? This would be a straightforward recipe and I think this would help to increase usage of WhatsGNU.

* The report has lots of information until you get to the "GNU score". I think the "GNU score" column should be the first column to draw the users attention directly to it.

* On lines 465,478, and 582 there are regex patterns that searches for "_GCA_". It would probably be best to standardize how strain names should be given instead of the current forced "_GCA_". Instead of, ">strain_GCA_12345.1_protein_2" as recommended something like ">GCA_12345|protein_2" at least then the regex could be replaced with a split on "|". It might even be worth adding a script or function to do this for the user. Or, another alternative would be to allow a directory of FASTAs with the "-m" option and do it automatically for the user at lines 179-183 in WhatsGNU.py (https://github.com/ahmedmagds/WhatsGNU/blob/master/WhatsGNU.py#L177-L183). Then the file name could just be added to the file.

*Should the output option "-o" also include the database files (pickle, text) and log file? Right now it outputs to the current working directory.

*WhatsGNU is very verbose (not a bad thing!), but users should have the option to quiet STDOUT. Looking into the code, I noticed WhatsGNU uses a print and logging statement for each of the outputs. Merging these into a single logging statement (maybe something like this: https://stackoverflow.com/questions/13733552/logger-configuration-to-log-to-file-and-print-to-stdout), could then allow users to quiet the STDOUT, but retain the logging feature.

* WhatsGNU has a few behaviors that fallback to a default value when the user messed something up. I think a better approach would to be to notify the users of these errors and exit. For example, users can not set the name of the database at creation WhatsGNU appends "_compressed_database_TIMESTR.txt" to the concatenated proteins file. This might be difficult to track in a script as to which database was used. Also, WhatsGNU does not check if a database already exists, it just creates another one. An outcome of the database existence check, would be that the '-m' and '-d' parameters could be merged into a single. But it is worth noting the database creation time is negligible so I found myself just using the '-m' parameter.

Another default behavior, when an output folder WhatsGNU creates a default results folder (./WhatsGNU_results_v1_{}/). I think this behavior, of circumventing user error, could be problematic for scripted analyses. A user may outputs to go to a specified directory, by default if the output directory exists the new directory is made in the current working directory. A better approach might be to say "output folder exists, will not overwrite unless --force (or something like that is used)". Also, this check happens late in program (WhatsGNU.py Line 362)

*There does not seem to be a simple way to programmatically download the pre-compressed databases stored on Google Drive using something like wget/curl. I had to download locally then upload to my server, losing the benefit of saving time by downloading a pre-compressed database. An alternative approach might be to include the NCBI Assembly accessions, then users could download them directly from NCBI and recreate the database themselves using something like https://github.com/kblin/ncbi-genome-download/. WhatsGNU builds the databases so quickly, I think an easier method for downloading precompressed databases would be necessary to make it useful. Also, for publication purposes, the precompressed databases files should have a version saved onto Zenodo/Figshare or some other similar service for historic purposes. Especially if the databases are going to be updated multiple times per year.

*Finally, it was a little disappointing that there were no plotting functions to allow users to create the plots found in Figure 1B,1C, and 1D. Also summary scripts should be included. Something to quickly give a overview of the results. Maybe consider an R wrapper?

**Reviewer 2**

The paper introduces a tool, WhatsGNU, as a means to quickly find out what's novel in a newly assembled microbial genome, especially where there are many strains already in databases. Use of the tool to find conserved versus non-conserved genes / proteins in a large number of genomes of the same species is another application which is explored. The paper is, in the most part, well written and seems to present a novel means to achieve its aims. However the report feels a little preliminary and it's not clear how strong the biological insights are which can be generated. I have a number of comments, as outlined below.

Major comments

- There is no detail in the paper as to how the GNU score is actually calculated. This needs to be made clear for the tool to be fully understood by its users.

- I'm not sure that the paper has fully explored or shown what is actually being measured by the GNU score. It is noted that there is an association between contig number in a genome and GNU scores. This suggests that the GNU scores may also be affected by protein size / length. Further, proteins encoded by genes containing homopolymer repeats, which are subject to issues in next-generation sequencing, might also end up with low GNU scores. A comprehensive analysis of what is ultimately reported as 'novel' (GNU score = 0) is required and how this works with respect to issues in genome sequencing and assembly. Would truncated genes / proteins have a GNU score of 0, too?

- The range of GNU scores (e.g. as shown in Fig 1C) and what the peaks in such graphs mean need to be fully explored and explained. What are the genes / proteins in the peaks? Are they sensible, in a biological sense? Are the ultra-conserved proteins what one might expect? Same question with the 'somewhat well-conserved' group of genes / proteins. Or is there something else that the GNU score is sensitive to which results in the peaks shown here (were the genomes sequenced and assembled in the same way?).

- Often, with these types of new tools, it's useful to compare the output with that of a traditional 'gold-standard' approach. Does an analysis such as that done in Figure 1C, or even Figure 1D if looking at a reduced set, match what one might see if benchmarked against a BLAST result set?

Minor comments

- p6, line 124. It's not clear what the experiment or analysis is here. More detail required.

- it looks like the tool has the capacity to generate a pan-proteome, which would be good. It would be useful for this to be done, and compared to a pan-genome analysis as a point of comparison.

**Response to reviewers**

**Reviewer 1**

WhatsGNU is a compression software designed to rapidly reduce genbank formatted proteomes to libraries of exact matches. Individual proteomes can be queried for the number of identical matches and hence a "novelty score" can be calculated. The results section is a series of vignettes of potential uses of the software, using moderately sized databases of S. aureus, M. tuberculosis and P. aeruginosa as examples. No novel scientific conclusions were drawn about the organisms as a result of these analyses.

1.In my hands the software installed easily and worked as advertised (see below). It was impressively fast - better performance than cd-hit, a software with similar applications. Downsides were the lack of natural support for custom databases and no built in function to generate summary tables and publication quality plots.

Response: The reviewer is entirely correct. We have now expanded our tool to handle custom databases using WhatsGNU_database_customizer.py and generate summary tables and publication quality plots using WhatsGNU_plotter.py. The plotter script generates heatmap, histograms, metadata percentage distribution bar plot and volcano plots.

2.Overall, I found this to be an interesting software but without a really great application. The major strength is allowing users a way to quickly get an idea of the proteome composition of a set of genomes from genbank. However, after doing this there is not much more that can be done with the WhatsGNU database. This is because of the principal limitation of the software - it can only return 100% matches, which means the database isn't a true pangenome representation and you would need to go on and run a software like ROARY on your data anyway. Other software exists to provide a "quick peek", such as mash (albeit with a completely different approach) but mash sketches can also be used for other applications such as calculating average nucleotide identity, building quick trees etc.

Response: WhatsGNU was designed to provide users with different answers than existing pangenome tools. It is a panallelome approach that identifies the protein allele frequency in the genomes of a species in public/private databases and associates different alleles with metadata (e.g. disease, clonal complex, etc..). We now present an updated version that also accepts pangenome analysis results from the Roary tool to link different alleles in the same orthologous group, and this is done only once at the database developmental stage. The user has the option to run WhatsGNU in its basic mode or ortholog mode (using the clustered proteins output file from Roary).

Minor/Specific Points:

3.This software should work on gene sequences as well as protein. Surprised that the authors did not mention this, as it’s a potential alternative for cgMLST.

Response: We agree and we added a sentence describing this potential application in line 125 (page 6). We are also considering a nucleotide-based feature along with other potential applications for future publications. However, we would expect nucleotide “novelty” to be much higher and also more complex, and have chosen to focus here on protein coding novelty.

4.Line 69 - Need to spell out what are different challenges on human versus bacterial proteomes. WhatsGNU itself is not limited to bacteria and could be used on human datasets.

Response: We agree. We have focused here on bacterial genomes because of the large numbers of sequences for some species and the relative heterogeneity of intraspecies diversity in bacteria. Future work will address non-microbial applications of this software. A brief note contextualizing our approach and the existing tools approach is now in line 73 (page 4) in the manuscript.

5.Line 81 - would be impressive to showcase WhatsGNU on the truly large datasets out there now - e.g 200,000+ Salmonella genomes.

Response: We have now tested the performance of WhatsGNU on 43,914 S. aureus genomes from the Staphopia database and on 216,642 Salmonella enterica genomes from the Enterobase database. The precompressed databases are available for the users to download and the compression statistics are provided in Figure 1B.

6.Line 109 - I think here you need to compare the strengths of WhatsGNU with mash, which is the tool most commonly used to answer this particular question at the moment.

Response: Thank you to the reviewer for this helpful suggestion. The comparison has been done and the results are very similar with minor changes in order and discussed in lines 125-127 (page 6) in the manuscript and in the supplementary file in the Mash comparison section.

7.Naming and Visibility:

I realize it is late in the game, but I think it might be worthwhile to reconsider the naming of "WhatsGNU". The naming is fun, but I think the usage of "GNU" is likely to confuse users. Is this tool a part of (or going to be) the GNU Project? If not, since GNU is a popular operating system and has many tools under its umbrella, I'm afraid WhatsGNU will never escape a false association with GNU. A Google search of GNU returns 100+ million results. Also, a quick Google search of "What's GNU" returns the board game "What's GNU": https://urldefense.proofpoint.com/v2/url?u=https-3A__www.amazon.com_Think- 2DFun-2D7760-2DWhats-2DGnu_dp_B00073JTQQ&d=DwIGaQ&c=vh6FgFnduejNhPPD0fl_yRaSfZy8CWbWnIf4XJhSqx8&r=CNNFLMxO4uNTPWvyDeZO8xce3yyjwBs48Y1sSH5FuaUPZzC66Xz_B36lEUoRyrh&m=A9IaOUXmANjW_02QO_0aRIG OWaZdjgvXmtF5f1HPzDQ&s=Affmm6zAmpVOE3Wh3cvg5OdBGoostlwh2xd4IPsUsH

Y&e=

As well as general posts regard what GNU is and what the GNU Project is. I was unsuccessful finding the WhatsGNU GitHub repo through Google search using terms like:

whatsgnu bioinformatics

whatsgnu github

whatsgnu gene novelty unit github

gene novelty unit github

But I am unsure how long the repo has been public. It may not have been indexed yet. Although, anything with "gnu" in the search was littered with results associated with GNU.

I did find this repo, https://urldefense.proofpoint.com/v2/url?u=https-

3A__github.com_mioux_whats-

2Dgnu&d=DwIGaQ&c=vh6FgFnduejNhPPD0fl_yRaSfZy8CWbWnIf4XJhSqx8&r=CNN

FLMxO4uNTPWvyDeZO8xce-

3yyjwBs48Y1sSH5FuaUPZzC66Xz_B36lEUoRyrh&m=A9IaOUXmANjW_02QO_0aRIG

OWaZdjgvXmtF5f1HPzDQ&s=yPspQyAl5sjhDZvpRw7Uqdx-

8zVs5U41CBrAY6NVmnM&e= , but it has been untouched for at least 4 years.

Response: We appreciate the reviewer’s concern, however at the time of the review the repository was new and that is why Google did not show our repository. Currently, search word “WhatsGNU” will bring up the repository as the second result while “WhatsGNU proteome” brings it up as a first result. As for the possible association with the GNU project, we believe that the name is different enough to escape this association, and we hope that our documentation makes that clear. As we have presented this application at conferences and to colleagues we have received a very positive response to the name.

8. Software remarks

*I followed installation information at

https://urldefense.proofpoint.com/v2/url?u=https-

3A__github.com_ahmedmagds_WhatsGNU&d=DwIGaQ&c=vh6FgFnduejNhPPD0fl_y

RaSfZy8CWbWnIf4XJhSqx8&r=CNNFLMxO4uNTPWvyDeZO8xce-

3yyjwBs48Y1sSH5FuaUPZzC66Xz_B36lEUoRyrh&m=A9IaOUXmANjW_02QO_0aRIG

OWaZdjgvXmtF5f1HPzDQ&s=h-

JKLU1WwpfB_OunKaR4E8YtOFWJYHIL5lA3ZOyxjVY&e=

Git clone, chmod 755, add to path all make sense and are pretty standard. I created a custom database based on 59 complete bacterial genomes. Everything completed quickly (~2seconds). A total of 100,960 proteins was compressed to 35,565 unique proteins. I received the expected report for the query. I created another database which included the 959 draft assemblies. This compressed 1,700,211 proteins into a database of 181,465 unique proteins in ~20 seconds. I then processed to query the 59 completed genomes against this new 959 sample database. Everything seemed to have finished successfully. I included "--topgenomes", "--hits", and "-- strainhits" in the execution. The "hits" files were created, the "topgenomes" and "strainhits" does not work for custom data. As "topgenomes" and "strainhits" is currently written it only works for strains with "_GCA_" in their name. I looked into the code, and it looks like "tophits" and "strainhits" will only work for GenBank assemblies (GCA) and not RefSeq assemblies (GCF) or custom assembly names. The authors acknowledge this problem but their solution is clumsy (attaching "GCA" to protein descriptions, and beyond the reach of many/most users). Currently this limits the functionality of WhatsGNU.

Response: This has been addressed using the new script in the tool WhatsGNU_database_customizer.py.

9.One thing to consider is if you call "WhatsGNU.py" without arguments it exits with a status 2. Makes sense since argparse recognizes `query_faa` was missing. A better approach might be to print the usage and exit with status code 0 if no arguments are given. Something like:

```

if len(sys.argv) == 1:

PARSER.print_help()

sys.exit(0)

ARGS = PARSER.parse_args()

```

Response: This has been changed accordingly in line 204 in the script.

10.Are there plans to submit this to Bioconda

(https://urldefense.proofpoint.com/v2/url?u=https-3A__bioconda.github.io_-29-F&d=DwIGaQ&c=vh6FgFnduejNhPPD0fl_yRaSfZy8CWbWnIf4XJhSqx8&r=CNNFLM

xO4uNTPWvyDeZO8xce-3yyjwBs48Y1sSH5FuaUPZzC66Xz_B36lEUoRyrh&m=A9IaOUXmANjW_02QO_0aRIG

OWaZdjgvXmtF5f1HPzDQ&s=h80sqgjtLgMYlGm5X0SeeTLs2Zh_BqtsU5SfxT_

Ooc&e= This would be a straightforward recipe and I think this would help to increase usage of WhatsGNU.

Response: We agree and a submission to Bioconda is in progress.

11.The report has lots of information until you get to the "GNU score". I think the "GNU score" column should be the first column to draw the users attention directly to it.

Response: We have updated the report to have the GNU score in the second column after the protein name.

12.On lines 465,478, and 582 there are regex patterns that searches for "_GCA_". It would probably be best to standardize how strain names should be given instead of the current forced "_GCA_". Instead of, ">strain_GCA_12345.1_protein_2" as recommended something like ">GCA_12345|protein_2" at least then the regex could be replaced with a split on "|". It might even be worth adding a script or function to do this for the user. Or, another alternative would be to allow a directory of FASTAs with the "-m" option and do it automatically for the user at lines 179-183 in WhatsGNU.py

(https://urldefense.proofpoint.com/v2/url?u=https-

3A__github.com_ahmedmagds_WhatsGNU_blob_master_WhatsGNU.py-23L177-

2DL183&d=DwIGaQ&c=vh6FgFnduejNhPPD0fl_yRaSfZy8CWbWnIf4XJhSqx8&r=CN

NFLMxO4uNTPWvyDeZO8xce-

3yyjwBs48Y1sSH5FuaUPZzC66Xz_B36lEUoRyrh&m=A9IaOUXmANjW_02QO_0aRIG

OWaZdjgvXmtF5f1HPzDQ&s=WwwfDiIzOKahS8yJWVMkiO3qQ_uDNY-

45Pqbp5P8sAM&e= ). Then the file name could just be added to the file.

Response: This has been addressed using the new script in the tool

WhatsGNU_database_customizer.py which handles FASTA files from different sources such as Prokka, GenBank, RefSeq and RAST. The script also handles gff files if the user decides to run Roary. The output of Roary in this case (clustered_proteins) will be compatible with WhatsGNU.

13.Should the output option "-o" also include the database files (pickle, text) and log file? Right now it outputs to the current working directory.

Response: This has been updated and all the generated files are included in the created results directory.

14.WhatsGNU is very verbose (not a bad thing!), but users should have the option to quiet STDOUT. Looking into the code, I noticed WhatsGNU uses a print and logging statement for each of the outputs. Merging these into a single logging statement

(maybe something like this: https://urldefense.proofpoint.com/v2/url?u=https-

3A__stackoverflow.com_questions_13733552_logger-2Dconfiguration-2Dto-2Dlog-

2Dto-2Dfile-2Dand-2Dprint-2Dto-

2Dstdout&d=DwIGaQ&c=vh6FgFnduejNhPPD0fl_yRaSfZy8CWbWnIf4XJhSqx8&r=CN

NFLMxO4uNTPWvyDeZO8xce-

3yyjwBs48Y1sSH5FuaUPZzC66Xz_B36lEUoRyrh&m=A9IaOUXmANjW_02QO_0aRIG

OWaZdjgvXmtF5f1HPzDQ&s=YSBOcVqOKR-iyp31yyYeaajvI8xl-mbUTAWEdlOqv-

Y&e= ), could then allow users to quiet the STDOUT, but retain the logging feature.

Response: We agree with the reviewer, and we have updated the script to have a quiet option that will quiet the STDOUT and keep the logging feature.

15.WhatsGNU has a few behaviors that fallback to a default value when the user messed something up. I think a better approach would to be to notify the users of these errors and exit. For example, users can not set the name of the database at creation WhatsGNU appends "_compressed_database_TIMESTR.txt" to the concatenated proteins file. This might be difficult to track in a script as to which database was used. Also, WhatsGNU does not check if a database already exists, it just creates another one. An outcome of the database existence check, would be that the '-m' and '-d' parameters could be merged into a single. But it is worth noting the database creation time is negligible so I found myself just using the '-m' parameter.

Response: We have updated the script to allow the user to assign a prefix for the compressed database.

16.Another default behavior, when an output folder WhatsGNU creates a default results folder (./WhatsGNU_results_v1_{}/). I think this behavior, of circumventing user error, could be problematic for scripted analyses. A user may outputs to go to a specified directory, by default if the output directory exists the new directory is made in the current working directory. A better approach might be to say "output folder exists, will not overwrite unless --force (or something like that is used)". Also, this check happens late in program (WhatsGNU.py Line 362)

Response: The script is now updated to warn the user if the output directory exists so the user can assign a new name or use the --force option to overwrite.

17.There does not seem to be a simple way to programmatically download the precompressed databases stored on Google Drive using something like wget/curl. I had to download locally then upload to my server, losing the benefit of saving time by downloading a pre-compressed database. An alternative approach might be to include the NCBI Assembly accessions, then users could download them directly from NCBI and recreate the database themselves using something like

https://urldefense.proofpoint.com/v2/url?u=https-3A__github.com_kblin_ncbi-

2Dgenome-

2Ddownload_&d=DwIGaQ&c=vh6FgFnduejNhPPD0fl_yRaSfZy8CWbWnIf4XJhSqx8&r

=CNNFLMxO4uNTPWvyDeZO8xce-

3yyjwBs48Y1sSH5FuaUPZzC66Xz_B36lEUoRyrh&m=A9IaOUXmANjW_02QO_0aRIG

OWaZdjgvXmtF5f1HPzDQ&s=PQoiktfL4O2O4pekiO5jab_xbbQALCbbCvP1PZEyFc8&

e= . WhatsGNU builds the databases so quickly, I think an easier method for downloading precompressed databases would be necessary to make it useful. Also, for publication purposes, the precompressed databases files should have a version saved onto Zenodo/Figshare or some other similar service for historic purposes. Especially if the databases are going to be updated multiple times per year.

Response: We have addressed this comment in two ways. First, we have now included a script “WhatsGNU_ get_GenBank_genomes.py” which will download the genomes of interest from GenBank using GCA number as contigs, protein faa or both. The precompressed databases are also now available through Dropbox which will make them available to download using wget with the instructions provided in the supplementary file. We will also use the Research Data Support service from SpringerNature to publish a permanent version of the compressed databases along with the scripts used in the publication.

18.Finally, it was a little disappointing that there were no plotting functions to allow users to create the plots found in Figure 1B,1C, and 1D. Also summary scripts should be included. Something to quickly give a overview of the results. Maybe consider an R wrapper?

Response: This has now been addressed using the WhatsGNU_plotter.py script which generates heatmap, histograms, metadata percentage distribution bar plot and Volcano plots. Examples are now shown in Figure 2B, C, D and E and supplementary figure 3.

**Reviewer 2**

The paper introduces a tool, WhatsGNU, as a means to quickly find out what's novel in a newly assembled microbial genome, especially where there are many strains already in databases. Use of the tool to find conserved versus non-conserved genes / proteins in a large number of genomes of the same species is another application which is explored. The paper is, in the most part, well written and seems to present a novel means to achieve its aims. However the report feels a little preliminary and it's not clear how strong the biological insights are which can be generated. I have a number of comments, as outlined below.

Response: We appreciate the time the reviewers spent reviewing the manuscript, and we hope that the new additions address the specific concerns about the preliminary aspects of the report. We now provide some examples of possible biological insights that can be garnered from WhatsGNU analysis.

Major comments

1.There is no detail in the paper as to how the GNU score is actually calculated. This needs to be made clear for the tool to be fully understood by its users.

Response: This is now more clearly explained in lines 61-72 (page 3-4) in the manuscript and in the WhatsGNU toolbox section in the supplementary materials.

2.I'm not sure that the paper has fully explored or shown what is actually being measured by the GNU score. It is noted that there is an association between contig number in a genome and GNU scores. This suggests that the GNU scores may also be affected by protein size / length. Further, proteins encoded by genes containing homopolymer repeats, which are subject to issues in next-generation sequencing, might also end up with low GNU scores. A comprehensive analysis of what is ultimately reported as 'novel' (GNU score = 0) is required and how this works with respect to issues in genome sequencing and assembly. Would truncated genes / proteins have a GNU score of 0, too?

Response: We agree with the reviewer that this was not clear at first, and we have attempted to clarify it in lines 111-119 (pages 5-6) in the manuscript by explaining that completely “novel” variants may in fact be due to sequencing errors or incomplete assemblies that produce truncated genes, and that GNU score of 0 should be treated cautiously with additional sequencing if needed. We have also further explained in the WhatsGNU toolbox section in the supplementary materials that the GNU score could be affected by the protein size if this increases the chances that any given position might mutate. Similarly, genes that contain homopolymer repeats or other regions that are vulnerable to NGS errors might also end up with lower GNU scores. To account for this limitation, we have also introduced the Ortholog Variant Rarity Index (OVRI) metric that takes into consideration the GNU score of other alleles in the same ortholog group. This metric would help to differentiate between ortholog groups with high levels of diversity or sequencing errors and others that are highly conserved. Truncated proteins would also be expected to have low or zero GNU scores if this truncation is not often seen in the database. Validated truncated protein sequences, would be very interesting to researchers since these may represent loss of function mutations.

3.The range of GNU scores (e.g. as shown in Fig 1C) and what the peaks in such graphs mean need to be fully explored and explained. What are the genes / proteins in the peaks? Are they sensible, in a biological sense? Are the ultra-conserved proteins what one might expect? Same question with the 'somewhat well-conserved' group of genes / proteins. Or is there something else that the GNU score is sensitive to which results in the peaks shown here (were the genomes sequenced and assembled in the same way?).

Response: We agree with the reviewer and we have attempted to explore these issues as much as possible in the space given for this short report. In the updated version of the tool the user will be provided with a text file with the genes in the novel and ultraconserved peaks, and the user has the option to adjust the cutoffs for this report. We have now also discussed briefly in the Figure 2C section and in the supplementary materials some of the proteins that are located in these peaks. Briefly, the novel peak contained some protein sequences of genes that might be expected to be more variable such as virulence proteins (eg., Staphostatin B and Clumping factor A), transcriptional regulators and IS elements. Appearing in the ultra-conserved peak proteins for critical cellular processes such as GpsB (Cell cycle protein), DivIC (Cell division protein), multiple 30S and 50S ribosomal proteins, SigA (RNA polymerase sigma factor) and ATP synthase subunits. We have also now plotted a histogram of the GNU scores of the isolate used in Figure 2C with gene ontology annotations (supplementary figure 2) to show the distribution of the biological processes hits associated with the proteins located in the novelty and ultra-conserved peaks. The plot did not show a striking pattern, however it showed that some accessory proteinsannotated with biological processes such as adhesion and aggregation are located in the novelty peak with low GNU scores while proteins involved in cellular processes and cellular component organization or biogenesis tend to be more conserved.

4.Often, with these types of new tools, it's useful to compare the output with that of a traditional 'gold-standard' approach. Does an analysis such as that done in Figure 1C, or even Figure 1D if looking at a reduced set, match what one might see if benchmarked against a BLAST result set?

Response: A small dataset was used to compare WhatsGNU results with blastp and it is provided in the Methods for Figure 1C (comparison with blastp) section in the supplementary file.

Minor comments

5.p6, line 124. It's not clear what the experiment or analysis is here. More detail required.

Response: This feature is now automated by WhatsGNU_plotter.py script and more details are provided in the Methods for Figure 2C section in the supplementary file.

6.It looks like the tool has the capacity to generate a pan-proteome, which would be good. It would be useful for this to be done, and compared to a pan-genome analysis as a point of comparison.

Response: We agree with the reviewer and we have added the ortholog mode to the tool which now would take the Roary (pangenome tool) output to link the different alleles in the same ortholog group. We see our tool as a complement to pangenome tools that offers a panallelomic approach and provides a measurement of how rare or widespread a specific protein allele is in the genomic database of a species.

**Second round**

**Reviewer 2**

Generally the paper is now improved and the authors have mostly addressed my set of comments from the initial review. The exception might be my comment 4, which I could have better phrased, whereby I had hoped that the authors would have compared the biological result from their tool with that which could be generated with blast P. The comparison done instead shows the speed of analysis from the two approaches (Fig 1D). The analysis given in 1D is nevertheless useful as it illustrates the speed of the whatsGNU tool, which is one of its features. The revision does give a bit more of an insight into the biological applications and possible insights that can be generated (FIg 2A is useful), which was my main concern with the first version. The supplementary figures / analyses that are now included to address some of my other comments are satisfactory and highlight the expected trends.

**Response to reviewers**

Generally the paper is now improved and the authors have mostly addressed my set of comments from the initial review. The exception might be my comment 4, which I could have better phrased, whereby I had hoped that the authors would have compared the biological result from their tool with that which could be generated with blast P. The comparison done instead shows the speed of analysis from the two approaches (Fig 1D).

We agree on the importance of this comparison.

We thought, in fact, that we had addressed this in our previous revised manuscript. In

addition to the performance comparison, we also compared the number of exact

matches of WhatsGNU and blastp, and they were identical. Methods and results for the

comparison are provided in the methods for Figure 1D (comparison with blastp) section

in the supplementary file. The dataset used to compare WhatsGNU with blastp and the

results are also available to download through Zenodo. The dataset is cited in the

reference list. We have also added this sentence “The number of exact matches

reported by WhatsGNU were identical to blastp (Additional files 1 and 4).” in the

manuscript to guide the reader for further information in the additional files.
